# Supplementary material for: Spot the bot: the inverse problems of NLP
Source: PeerJ Comput Sci. 2024 Dec 9;10:e2550. doi: 10.7717/peerj-cs.2550 (PMC11784749; doi:10.7717/peerj-cs.2550)
Supplement: Supplemental Information 1 — The middle top area includes chaotic processes, the right lower corner - white noise, the left lower corner - regular oscillations, the area in the middle - coloured noise. From Gromov & Dang (2023b). [file peerj-cs-10-2550-s001.pdf]

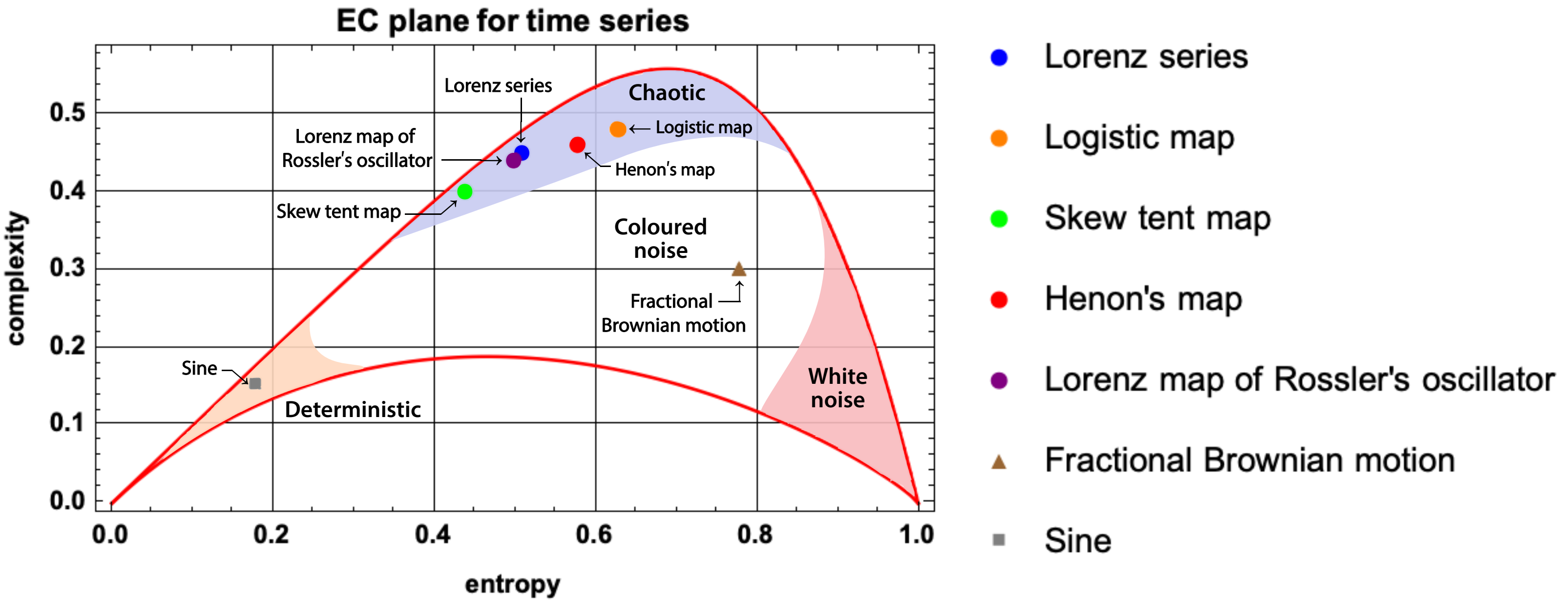
EC plane for time series
complexity
entropy
0.0
0.1
0.2
0.3
0.4
0.5
0.0
0.2
0.4
0.6
0.8
1.0
Chaotic
Lorenz series
Logistic map
Lorenz map of
Rossler's oscillator
Henon's map
Skew tent map
Coloured
noise
Fractional
Brownian motion
Sine
Deterministic
White
noise
Lorenz series
Logistic map
Skew tent map
Henon's map
Lorenz map of Rossler's oscillator
Fractional Brownian motion
Sine
