# Supplementary figures and images for: Spot the bot: the inverse problems of NLP
Source: PeerJ Comput Sci. 2024 Dec 9;10:e2550. doi: 10.7717/peerj-cs.2550 (PMC11784749; doi:10.7717/peerj-cs.2550)

# Russian

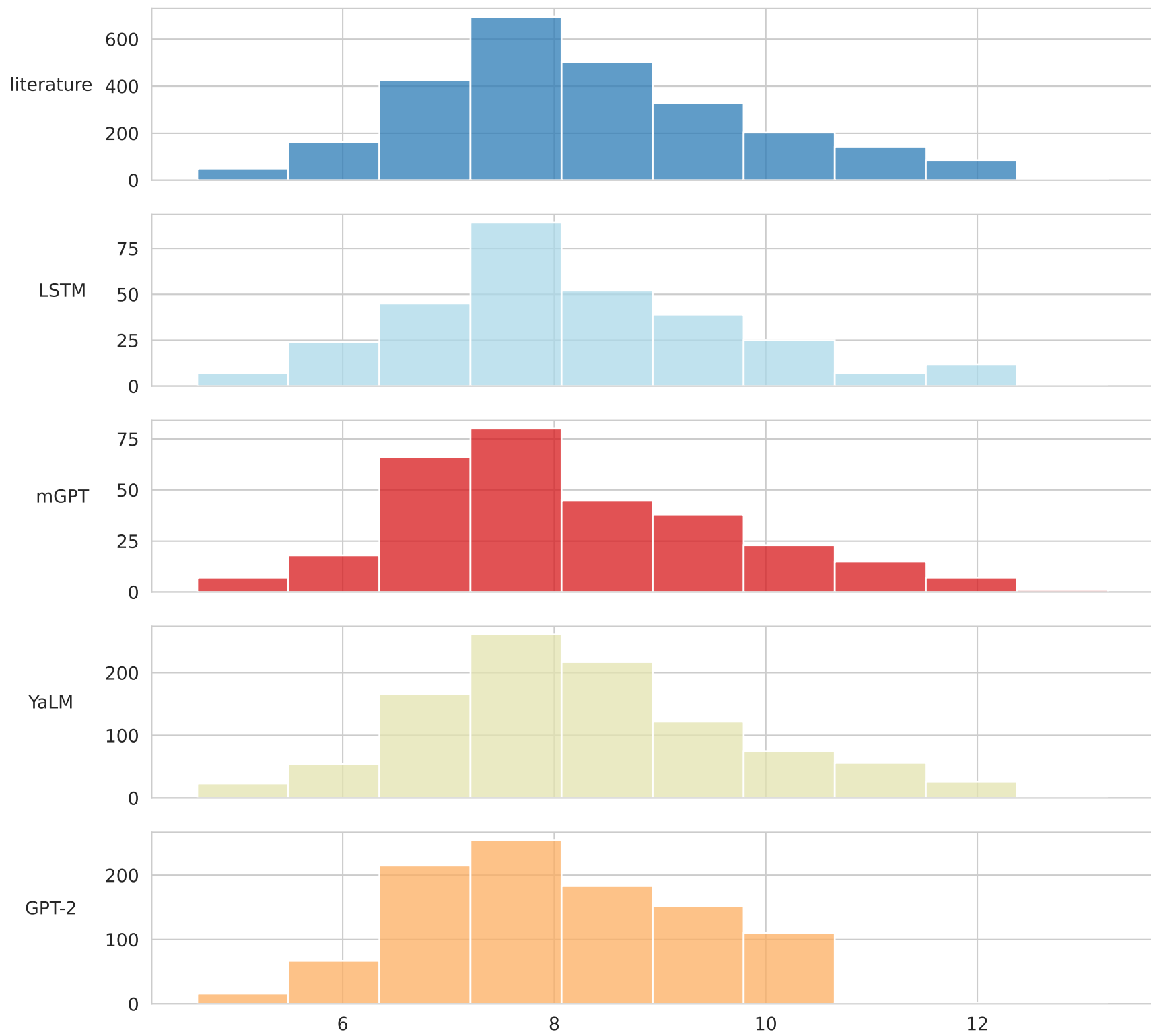

Supplement: Supplemental Information 3 [file peerj-cs-10-2550-s003.pdf]

English

German

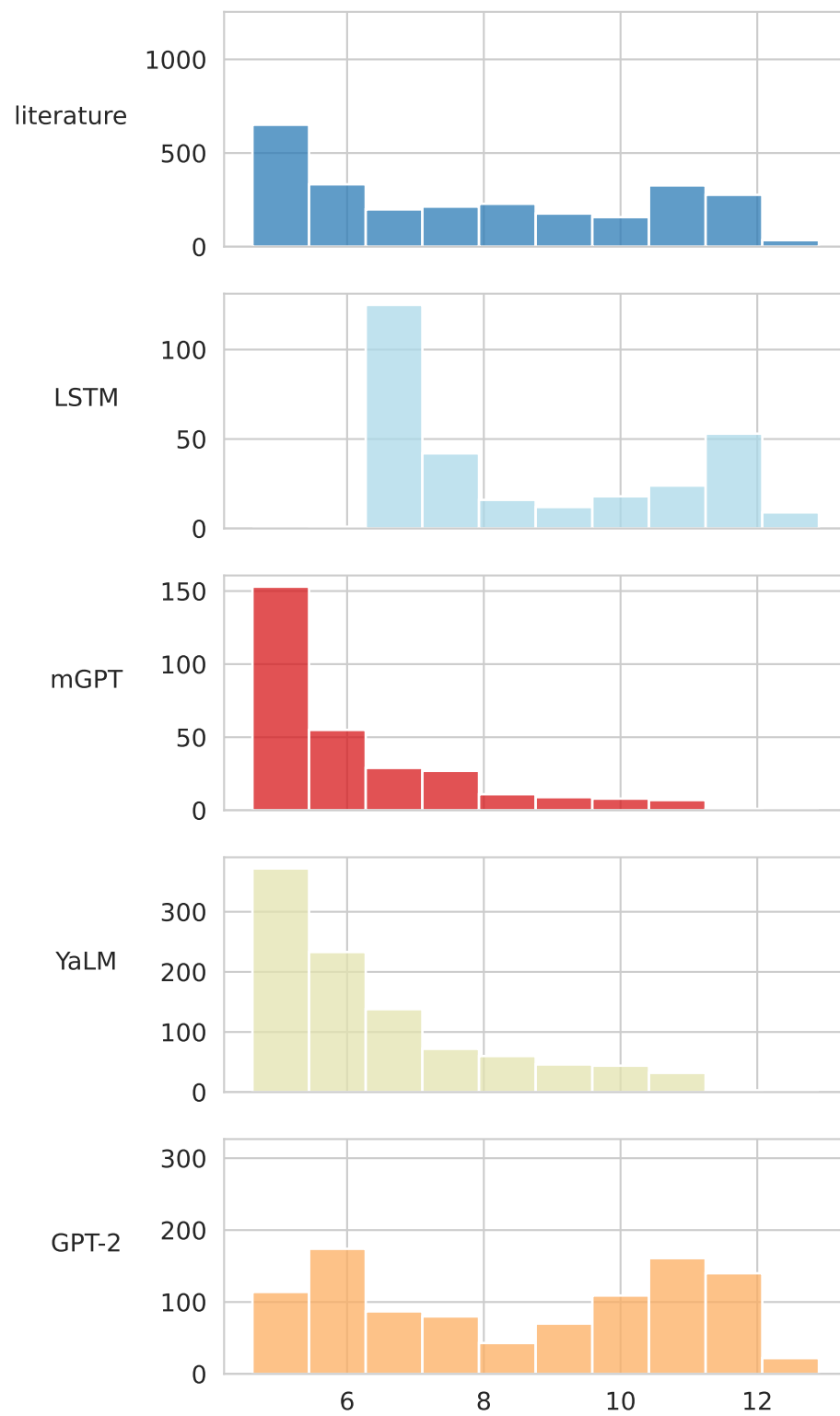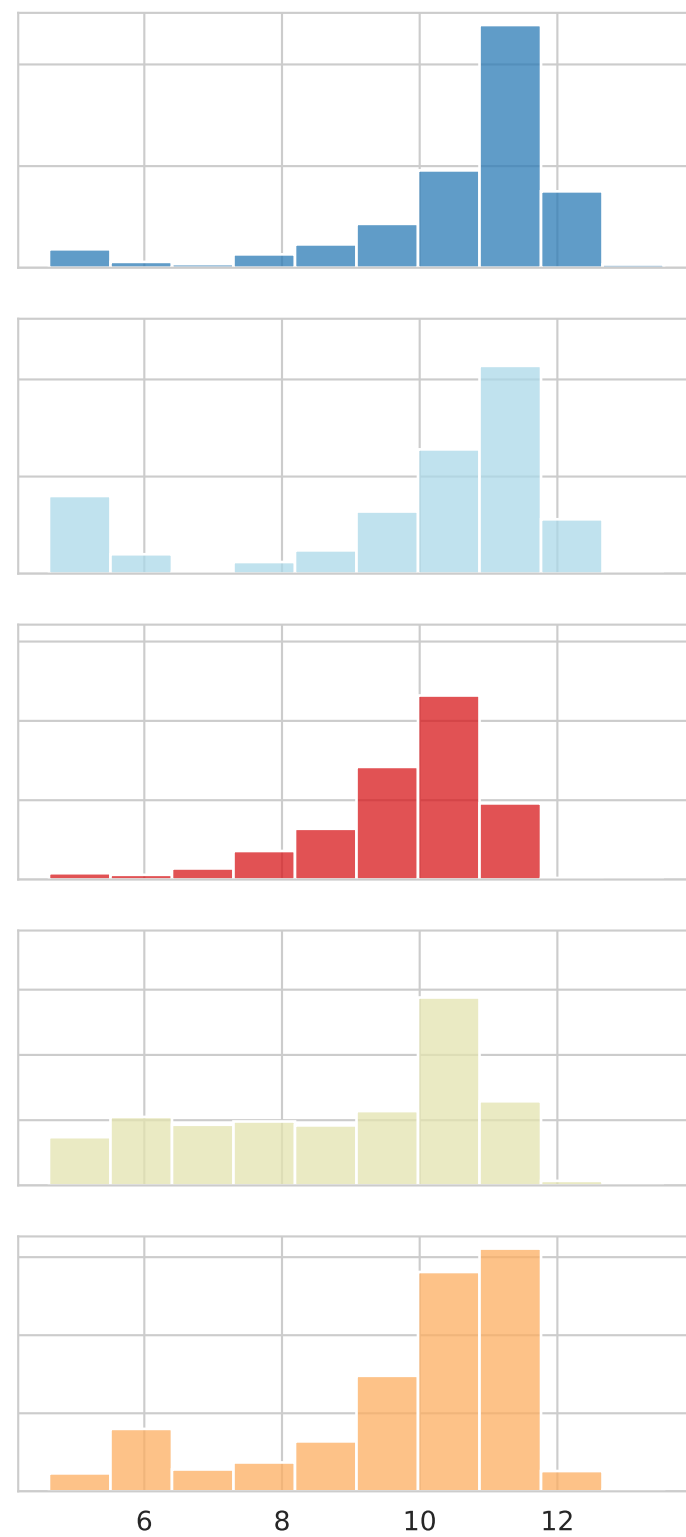

Supplement: Supplemental Information 4 [file peerj-cs-10-2550-s004.pdf]

French

Vietnamese

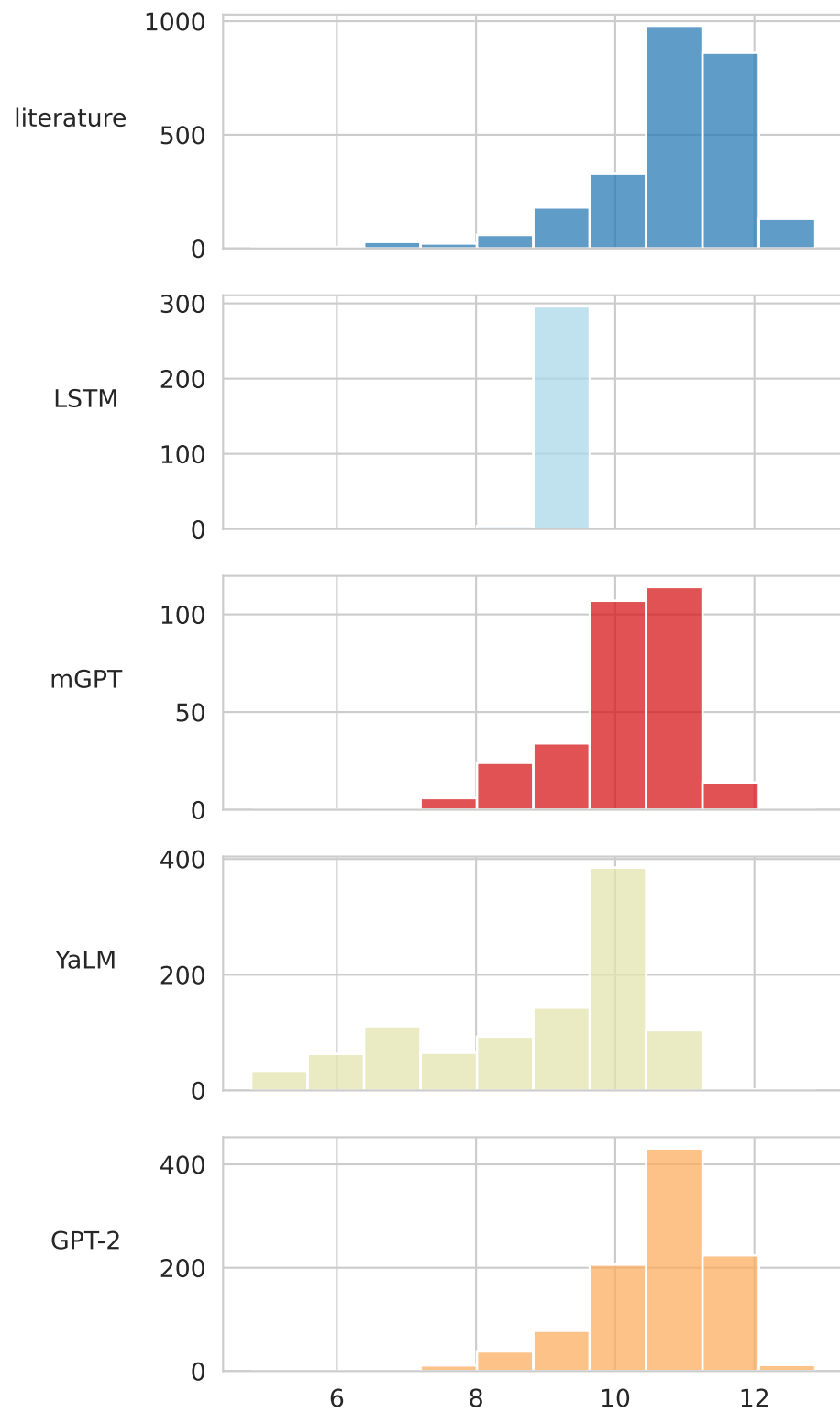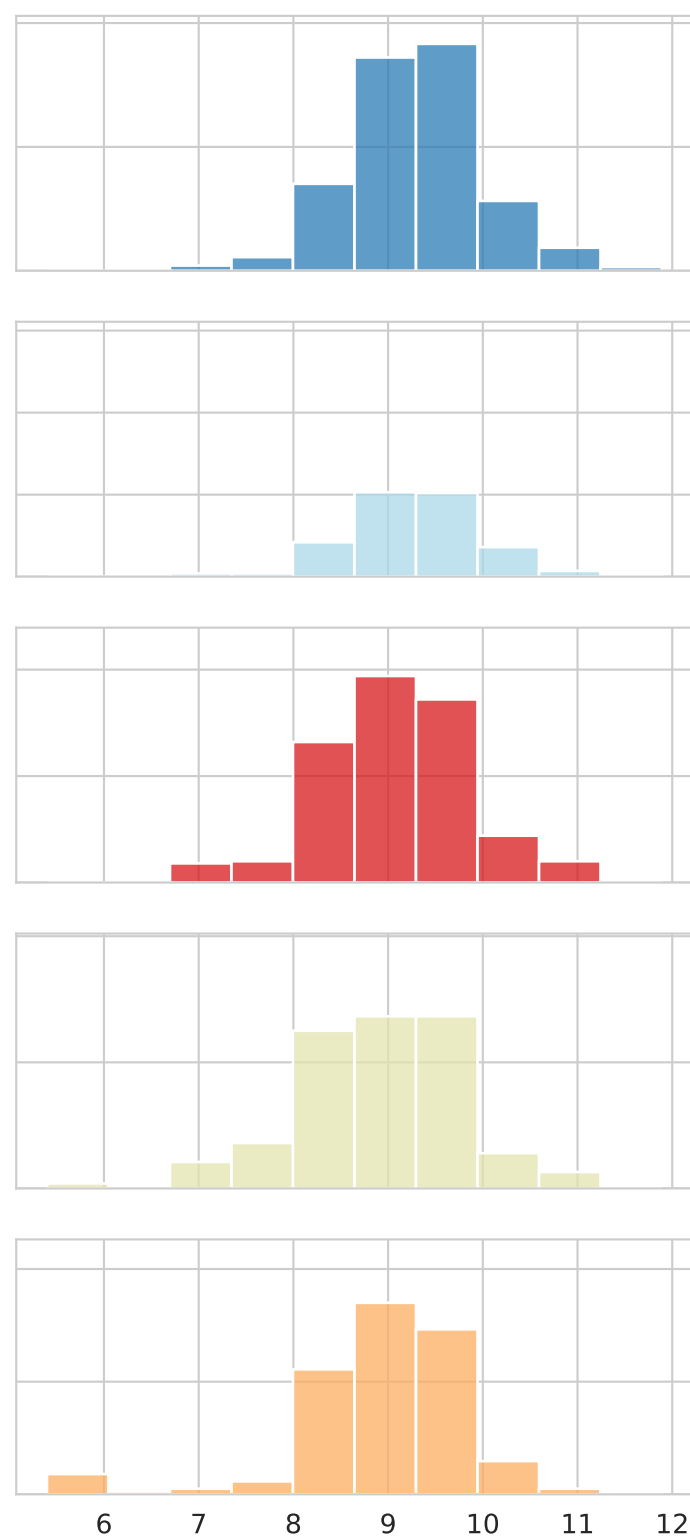

Supplement: Supplemental Information 5 [file peerj-cs-10-2550-s005.pdf]
