# Supplementary material for: Spot the bot: the inverse problems of NLP
Source: PeerJ Comput Sci. 2024 Dec 9;10:e2550. doi: 10.7717/peerj-cs.2550 (PMC11784749; doi:10.7717/peerj-cs.2550)
Supplement: Supplemental Information 12 [file peerj-cs-10-2550-s012.docx]

| Language | Hugging Face model | # parameters |
| --- | --- | --- |
| Russian | ai-forever/rugpt3large_based_on_gpt2 | 760M |
| English | openai-community/gpt2 | 124M |
| German | dbmdz/german-gpt2 | 124M |
| Vietnamese | NlpHUST/gpt2-vietnamese | 124M |
| French | dbddv01/gpt2-french-small | 124M |

**Table 2. Information about used GPT-2 models**
