# Supplementary material for: Spot the bot: the inverse problems of NLP
Source: PeerJ Comput Sci. 2024 Dec 9;10:e2550. doi: 10.7717/peerj-cs.2550 (PMC11784749; doi:10.7717/peerj-cs.2550)
Supplement: Supplemental Information 13 [file peerj-cs-10-2550-s013.docx]

|  | LSTM | GPT-2 | mGPT | YaLM |
| --- | --- | --- | --- | --- |
| Russian | 12185 | 6287 | 13097 | 12405 |
| English | 36721 | 32682 | 2807 | 3953 |
| German | 56423 | 42439 | 30650 | 23252 |
| French | 10813 | 55387 | 33897 | 16236 |
| Vietnamese | 13475 | 10460 | 11479 | 10105 |

**Table 3. Average generated text length (in tokens)**
