# Supplementary material for: Spot the bot: the inverse problems of NLP
Source: PeerJ Comput Sci. 2024 Dec 9;10:e2550. doi: 10.7717/peerj-cs.2550 (PMC11784749; doi:10.7717/peerj-cs.2550)
Supplement: Supplemental Information 14 — SVM, Support Vector Machine; DT, Decision Tree; RF, Random Forest. [file peerj-cs-10-2550-s014.docx]

|  | Russian | English | German | French | Vietnamese |
| --- | --- | --- | --- | --- | --- |
| SVM | **0.88** | 0.64 | 0.97 | **0.96** | 0.95 |
| DT | 0.77 | 0.82 | 0.97 | 0.64 | 0.95 |
| RF | 0.78 | **0.83** | **0.98** | 0.87 | **0.97** |

**Table S1. Accuracy score values for classification with entropy-complexity measures. SVM stands for Support Vector Machine; DT, for Decision Tree; RF, for Random Forest.**
