# Supplementary material for: Spot the bot: the inverse problems of NLP
Source: PeerJ Comput Sci. 2024 Dec 9;10:e2550. doi: 10.7717/peerj-cs.2550 (PMC11784749; doi:10.7717/peerj-cs.2550)
Supplement: Supplemental Information 15 [file peerj-cs-10-2550-s015.docx]

|  | Russian | English | German | French | Vietnamese |
| --- | --- | --- | --- | --- | --- |
| Support Vector Machine | | | | | |
| SVD | 0.54 | 0.42 | 0.59 | **0.74** | 0.50 |
| CBOW | 0.50 | **0.63** | **0.77** | 0.50 | **0.73** |
| Skip-Gram | 0.50 | 0.50 | 0.65 | 0.71 | 0.63 |
| Decision Tree | | | | | |
| SVD | **0.61** | **0.78** | 0.58 | **0.85** | **0.67** |
| CBOW | 0.50 | 0.58 | **0.81** | 0.50 | 0.59 |
| Skip-Gram | 0.50 | 0.50 | 0.58 | 0.74 | 0.60 |
| Random Forest | | | | | |
| SVD | **0.64** | **0.79** | 0.68 | **0.86** | 0.67 |
| CBOW | 0.51 | 0.61 | **0.82** | 0.50 | **0.68** |
| Skip-Gram | 0.50 | 0.50 | 0.63 | 0.78 | 0.62 |

**Table S2. Accuracy score values for classification with semantic trajectory characteristics.**
