# Supplementary material for: Spot the bot: the inverse problems of NLP
Source: PeerJ Comput Sci. 2024 Dec 9;10:e2550. doi: 10.7717/peerj-cs.2550 (PMC11784749; doi:10.7717/peerj-cs.2550)
Supplement: Supplemental Information 16 [file peerj-cs-10-2550-s016.docx]

|  | Russian | English | German | French | Vietnamese |
| --- | --- | --- | --- | --- | --- |
| Support Vector Machine | | | | | |
| SVD | 0.60 | 0.75 | **0.91** | 0.93 | 0.57 |
| CBOW | 0.68 | **0.88** | 0.69 | 0.69 | 0.80 |
| Skip-Gram | **0.71** | 0.82 | 0.69 | **0.95** | **0.84** |
| Decision Tree | | | | | |
| SVD | 0.68 | 0.74 | **0.88** | **0.86** | **0.80** |
| CBOW | **0.84** | **0.80** | 0.75 | 0.69 | 0.59 |
| Skip-Gram | 0.73 | 0.73 | 0.70 | 0.74 | 0.58 |
| Random Forest | | | | | |
| SVD | 0.65 | 0.80 | **0.90** | 0.65 | **0.64** |
| CBOW | **0.84** | **0.85** | 0.73 | 0.70 | 0.58 |
| Skip-Gram | 0.82 | 0.80 | 0.67 | **0.74** | 0.59 |

**Table S3. Accuracy score values for Wishart clustering-based classifiers.**
