# Supplementary material for: Spot the bot: the inverse problems of NLP
Source: PeerJ Comput Sci. 2024 Dec 9;10:e2550. doi: 10.7717/peerj-cs.2550 (PMC11784749; doi:10.7717/peerj-cs.2550)
Supplement: Supplemental Information 17 [file peerj-cs-10-2550-s017.docx]

|  | Russian | English | German | French | Vietnamese |
| --- | --- | --- | --- | --- | --- |
| Support Vector Machine | | | | | |
| SVD | 0.70 | 0.72 | **0.91** | 0.58 | **0.69** |
| CBOW | 0.55 | **0.95** | 0.84 | **0.86** | 0.62 |
| Skip-Gram | 0.62 | 0.86 | 0.86 | **0.86** | 0.56 |
| Decision Tree | | | | | |
| SVD | 0.60 | 0.82 | **0.88** | 0.59 | **0.77** |
| CBOW | 0.79 | **0.87** | 0.65 | 0.64 | 0.57 |
| Skip-Gram | **0.86** | 0.84 | 0.59 | **0.77** | 0.55 |
| Random Forest | | | | | |
| SVD | 0.66 | **0.92** | **0.88** | 0.85 | **0.74** |
| CBOW | 0.55 | 0.90 | 0.61 | **0.93** | 0.54 |
| Skip-Gram | **0.82** | 0.87 | 0.72 | 0.73 | 0.56 |

**Table S4. Accuracy score values for K-Means clustering-based classifiers.**
