# Supplementary material for: Spot the bot: the inverse problems of NLP
Source: PeerJ Comput Sci. 2024 Dec 9;10:e2550. doi: 10.7717/peerj-cs.2550 (PMC11784749; doi:10.7717/peerj-cs.2550)
Supplement: Supplemental Information 18 [file peerj-cs-10-2550-s018.docx]

|  | Russian | English | German | French | Vietnamese |
| --- | --- | --- | --- | --- | --- |
| Support Vector Machine | | | | | |
| Wishart | 0.59 | 0.63 | 0.50 | 0.50 | **0.67** |
| Fuzzy Wishart | 0.49 | 0.66 | 0.50 | **0.88** | 0.60 |
| K-Means | 0.50 | **0.80** | 0.51 | 0.63 | 0.65 |
| C-Means | **0.92** | 0.75 | 0.52 | 0.47 | 0.54 |
| Decision Tree | | | | | |
| Wishart | 0.56 | 0.71 | 0.72 | 0.64 | 0.65 |
| Fuzzy Wishart | 0.70 | 0.85 | **0.86** | **0.92** | **0.88** |
| K-Means | **0.97** | **0.86** | 0.63 | 0.70 | 0.67 |
| C-Means | 0.93 | 0.78 | 0.68 | 0.64 | 0.73 |
| Random Forest | | | | | |
| Wishart | 0.55 | 0.73 | 0.71 | 0.61 | 0.67 |
| Fuzzy Wishart | 0.70 | 0.85 | **0.89** | **0.93** | **0.81** |
| K-Means | **0.98** | **0.87** | 0.61 | 0.51 | 0.70 |
| C-Means | 0.95 | 0.78 | 0.60 | 0.67 | 0.72 |

**Table S5. Accuracy score values for classifiers based on intra-cluster distances.**
