# Supplementary material for: Spot the bot: the inverse problems of NLP
Source: PeerJ Comput Sci. 2024 Dec 9;10:e2550. doi: 10.7717/peerj-cs.2550 (PMC11784749; doi:10.7717/peerj-cs.2550)
Supplement: Supplemental Information 19 — SVM, Support Vector Machine; DT, Decision Tree; RF, Random Forest. [file peerj-cs-10-2550-s019.docx]

|  | Russian | English | German | French | Vietnamese |
| --- | --- | --- | --- | --- | --- |
| SVM | 0.82 | **0.98** | 0.63 | 0.82 | **0.74** |
| DT | 0.98 | 0.88 | 0.90 | **0.86** | 0.72 |
| RF | **0.99** | 0.91 | **0.91** | **0.86** | 0.72 |

**Table S6. Accuracy score values for unified classification model. SVM stands for Support Vector Machine; DT, for Decision Tree; RF, for Random Forest.**
